# *In vivo* quantification of the secretion rates of the hemolysin A Type I secretion system

**DOI:** 10.1038/srep33275

**Published:** 2016-09-12

**Authors:** Michael H. H. Lenders, Tobias Beer, Sander H. J. Smits, Lutz Schmitt

**Affiliations:** 1 Institute of Biochemistry, Heinrich-Heine-Universitaet, 40225 Duesseldorf, Germany

## Abstract

Type 1 secretion systems (T1SS) of Gram-negative bacteria secrete a broad range of substrates into the extracellular space. Common to all substrates is a C-terminal secretion sequence and nonapeptide repeats in the C-terminal part that bind Ca^2+^ in the extracellular space, to trigger protein folding. Like all T1SS, the hemolysin A (HlyA) T1SS of *Escherichia coli* consists of an ABC transporter, a membrane fusion protein and an outer membrane protein allowing the one step translocation of the substrate across both membranes. Here, we analyzed the secretion rate of the HlyA T1SS. Our results demonstrate that the rate is independent of substrate-size and operates at a speed of approximately 16 amino acids per transporter per second. We also demonstrate that the rate is independent of the extracellular Ca^2+^ concentration raising the question of the driving force of substrate secretion by T1SS in general.

Many Gram-negative bacteria especially pathogens have evolved dedicated secretion systems to translocate virulence factors into the extracellular medium or directly into the host cell^1^. Among these nanomachineries, Type 1 secretion systems (T1SS) are the most simple systems as they consist of an ATP-binding cassette (ABC) transporter, a membrane fusion protein (MFP), both located in the inner membrane (IM), and an outer membrane protein (OMP). T1SS are able to transport a rather diverse group of proteins with different functions^2^, for example hemophores such as HasA (188 amino acids, 19 kDa) from *S. marcescens*^3^, lipases such as LipA (613 amino acids, 65 kDa) from *S. marcescens*^2^, adenylaste cyclases like CyaA (1706 amino acids, 177 kDA) from *Bordetella pertussis*^2^, proteases like alkaline protease (479 amino acids, 50 kDA) from *Pseudomonas aeruginosa*^2^, pore-forming toxins like hemolysin A (1024 amino acids, 110 kDa) from *E. coli*^2^ or large adhesion factors such as LapA (8682 amino acids, ~900 kDa) from *P. fluorescens*^4^. Common to all these substrates is the presence of a secretion signal that is encoded within the C terminus that in contrast to other secretion systems is not cleaved during or after secretion. In the case of HlyA the last 50 to 60 C-terminal amino acids represent this sequence, which is essential and sufficient for the secretion process^5^,^6^,^7^,^8^,^9^.

One prominent family of T1SS substrates is the repeats in toxin (RTX) proteins. Characteristic for these proteins are glycine-rich, nonapeptide repeats (GG repeats) with the consensus sequence GGxGxDxUx (where x can be any amino acid and U is a large or hydrophobic amino acid)^10^ that are also located in the C-terminal part of the protein, just upstream of the secretion signal. The number of these repeats depends on the size of the protein with on average one repeat for every 6–12 kDa^2^,^10^.

The paradigm of T1SS that translocates RTX toxins is the hemolysin A secretion machinery found in uropathogenic *E. coli* strains. HlyA is a 1024 amino acid (110 kDa) pore-forming toxin, which harbors six conserved GG repeats^11^. The HlyA T1SS is composed of the ABC transporter hemolysin B (HlyB), the MFP hemolysin D (HlyD) and TolC, the endogenously expressed OMP.

Recently, it was demonstrated that HlyA is secreted in an unfolded form and that the folding rate of the passenger dictates the efficiency of secretion^12^. In the extracellular space, Ca^2+^ binds to the GG repeats with an affinity of approximately 100 μM^13^,^14^,^15^,^16^ and triggers folding of HlyA or the secreted substrate in general^16^. Since the intracellular Ca^2+^ concentration in the cytosol of *E. coli* is roughly 300 nM, HlyA and RTX proteins remain unfolded^16^,^17^. The binding of Ca^2+^ (concentration of up to 10 mM in the extracellular space^17^) and the subsequent folding of HlyA was consequently proposed to act as driving or ‘pulling’ force for secretion, acting as a ratchet^14^,^18^. However, experimental evidence supporting such a mechanism has only been reported recently for the adenylate cyclase toxin CyaA from *Bordetella pertussis*^19^.

Furthermore, fusions using a fast folding variant of GFP (eGFP) upstream of HlyA or a C-terminal fragment of HlyA (HlyAc) demonstrated that transport is unidirectional with the C-terminal secretion signal appearing first at the cell surface^20^. HlyAc consists of the last 218 amino acids of HlyA and contains three conserved GG repeats in addition to the secretion sequence. The secretion of HlyAc is as efficient as HlyA, but the protein is more stable and more efficiently purified^12^.

It is currently assumed for type 1 secretion that the substrate interacts with the IM components and recruits the OMP to form a continuous tunnel-channel across the periplasmic space. Subsequently, the substrate is transported in an unfolded, secretion-competent state in one step from the cytosol to the extracellular space. Energy is provided through the ABC transporter^21^ and through the proton motif force, which was reported to be essential at an early stage of the secretion process^22^.

The secretion rate of hemolysin A as well as for any other type 1 protein has not been experimentally determined. However, in the case of the SipA Type 3 secretion system (T3SS), secretion varied between 7–60 molecules SipA per sec per cell, as determined by time-lapse fluorescence spectroscopy^23^. As the number of T3SS translocons per cell is not known in this case, a precise secretion rate cannot be deduced. In the case of SecA-dependent transport mediated by SecYEG translocon, the rate limiting step, ATP hydrolysis, was determined experimentally *in vitro*^24^. Assuming that one ATP molecule energizes the translocation of 20–30 amino acids^25^,^26^, a translocation rate of 152–228 amino acids per sec per transporter can be calculated.

To address the question of the secretion rate, we used the concept of a stalled T1SS to quantify the amount of active HlyA T1SS per *E.* coli cell^20^. This allowed us to calculate the secretion rate of the entire HlyA, or the C-terminal fragment HlyAc. Our data demonstrate that the number of GG repeats has no influence on the secretion rate per amino acid and per transporter, that Ca^2+^ does not influence the secretion rate, and that ATP hydrolysis is necessary for substrate exposure at the extracellular cell surface.

## Results

### Determination of the amount of active T1SS

**Figure 1:**
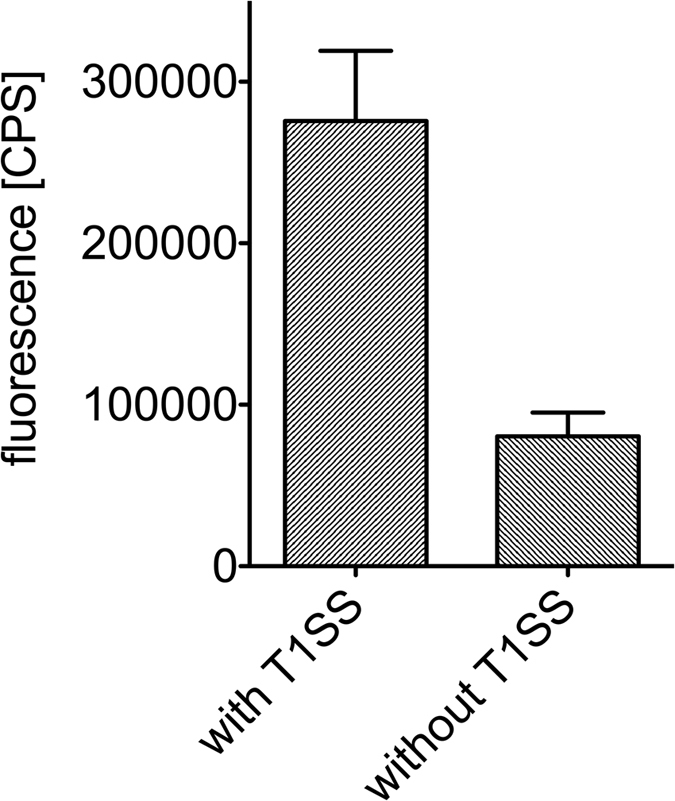
Total cell fluorescence of Cy3. Immunofluorescent labeled *E. coli* cells expressing eGFP-HlyAc in the presence (left bar) and absence (right bar) of HlyB and HlyD, respectively. Error bars represent the standard deviation of the Cy3 fluorescence of at least three biological replicates.

The prerequisite for quantification of the substrate secretion rate of any T1SS is an accurate determination of the number of active T1SS translocons per cell. Here, we applied the concept of stalled T1SS complexes that was previously used to determine the directionality of substrate translocation^20^. Accordingly, an eGFP-HlyAc fusion construct was employed to stall the HlyA T1SS within the membrane^20^. In the stalled complex, the C-terminal part of the fusion protein, i.e. HlyAc, is exposed at the cell surface, while eGFP remains in the cytosol and plugs the translocation pore^20^. The extracellular exposed HlyAc was visualized on the cell surface using an HlyA specific, polyclonal antibody, in combination with a secondary antibody labeled with a Cy3 fluorophore (See Fig. 3 of ref. 20). Since the nonspecific binding of both antibodies, anti-HlyA and the Cy3-labeled secondary antibody, is not significant (Fig. 1), these antibodies possess the major advantage that binding to secreted HlyA or parts of HlyA visualizes only active and correctly assembled secretion machineries, that consist of both HlyB and HlyD. We made the assumption that on average one HlyA primary antibody binds to each surface exposed HlyAc and on average one Cy3-labeled secondary antibody binds per primary HlyA antibody. Cells without HlyB and HlyD cannot expose an HlyAc fragment at the cell surface and therefore represent background fluorescence (Fig. 1, right bar, 8.05 × 10^5^ ± 1.48 × 10^5^ CPS), which was subtracted from every measurement. The amount of specific fluorescence observed for HlyA-exposed at the surface of *E. coli* is therefore 1.95 × 10^5^ ± 4.59 × 10^4^ CPS. To calculate the amount of bound antibody a regression analysis was performed (Supplementary Fig. 1). The intensity of the fluorescence signal was multiplied by the slope of the calibration line resulting in the exact amount of active and stalled translocons present in the *E. coli* membrane, i.e. 0.3 pmol (1.80 × 10^11^ molecules). Dividing the number of T1SSs by the number of cells (4 × 10^7^ cells in 50 μL solution of an OD_600_ of 1.0) resulted in an average of 4509 ± 1061 active T1SS cell^−1^. To validate the determined value, we used a second, commercially available, secondary Cy3-labeled antibody. Following the same experimental procedure, including an antibody specific calibration line (Supplementary Fig. 1), we determined the average number of active HlyA translocons to be 4554 ± 1616 per cell. This value is in excellent agreement with the result of the first Cy3-labeled secondary antibody.

A second method to determine the amount of HlyB in the membrane was applied, namely Western blotting. Cells secreting HlyAc were solubilized, separated by SDS-PAGE and analyzed using a polyclonal HlyB antibody to quantify the HlyB signal by comparison of the signal intensity of a dilution series of purified HlyB of known concentration, which was included on the same Western blot. The signal intensity of the different concentrations of purified HlyB was used to calculate a calibration line (slope: 1.26 × 10^−16^ mol; Supplementary Fig. 2). This calibration line was subsequently used to determine the number of HlyB molecules present in cells secreting HlyAc or HlyA. Thereby, the determined signal intensity of HlyB within cells secreting HlyA corresponded to 11 291 ± 1018, which is equal to 1.42 ± 0.13 pmol HlyB. Divided by the number of cells (8 × 10^7^ cells), this corresponds to 10 710 ± 965 molecules of HlyB per cell. Since HlyB is a functional dimer^27^, 5355 ± 483 functional units are present per cell. Thus, the Western blot analysis puts an upper limit on the number of T1SS per cell. The results of the fluorescence analysis of the stalled T1SS are in accordance with this upper limit and indicate that nearly all HlyB molecules are recruited to the T1SS.

In summary, the concept of a stalled T1SS allowed us to determine the number of active secretion machineries per cell and resulted in an average value of 4532 ± 966 T1SS cell^−1^, which was used in the subsequent analysis (Supplementary Table 1).

### Quantifying the secretion rate of HlyA and HlyAc

The determined number of T1SS cell^−1^ provided us with the possibility to quantify the secretion rate for HlyA and HlyAc, respectively both are equally well secreted by the HlyA T1SS^2^,^28^. Furthermore and as described by Lenders *et al*.^20^, substrate is always present in the cytosol of *E. coli* during secretion. This indicates that the secretion of HlyA is the rate-limiting step and not its synthesis.

For the purpose of quantification of the secretion rate, HlyA was secreted for four hours and samples of the supernatant were taken every hour. In parallel, cell growth was monitored each hour during secretion by measuring the OD at 600 nm. Based on the optical density, the total number of cells was calculated. Samples of the supernatant and a dilution series of purified HlyA of known concentration were analyzed by SDS-PAGE. The integrated signal intensity of the samples of purified HlyA at different concentrations was used to generate a regression line. This line allowed the quantification of secreted HlyA over time. Here, the integrated signal intensity of 1.09 × 10^4^ corresponds to 9.6 pmol HlyA (5.781 × 10^12^ molecules). Knowledge of the amount of secreted HlyA at more than one time point enabled us to determine the secretion level of HlyA per time. Here, a value of 0.13 pmol min^−1^ (7.83 × 10^10^ HlyA min^−1^). Divided by the number of cells per time (2.03 × 10^7^ cells min^−1^), this number corresponds to a value of 3856 secreted HlyA molecules in cell^−1^ min^−1^. If one takes the number of amino acids of HlyA (1024 aa) into account, the calculated secretion rate of HlyA per T1SS and per cell (4532), per sec was 14.5 amino acids (T1SS^−1^ s^−1^) in this particular example. During the time period of the secretion experiments, HlyA molecules were still present in the cytosol indicating that not the synthesis of HlyA but the translocation step of the substrate is the rate-limiting factor.

The number of cells and the number of secreted HlyA in the exponential cell growth phase of *E. coli* were used for calculation of the secretion rate. This phase is normally present within the first two hours after induction. Supplementary Tables 2 and 3 summarize all values for secreted HlyA and HlyAc, respectively, for the secretion experiments performed for 2 h and 4 h, respectively, the corresponding HlyA secretion level [mol min^−1^], cell growth [cells min^−1^] and the final secretion rate of HlyA and HlyAc [aa T1SS^−1^ s^−1^].

**Figure 2:**
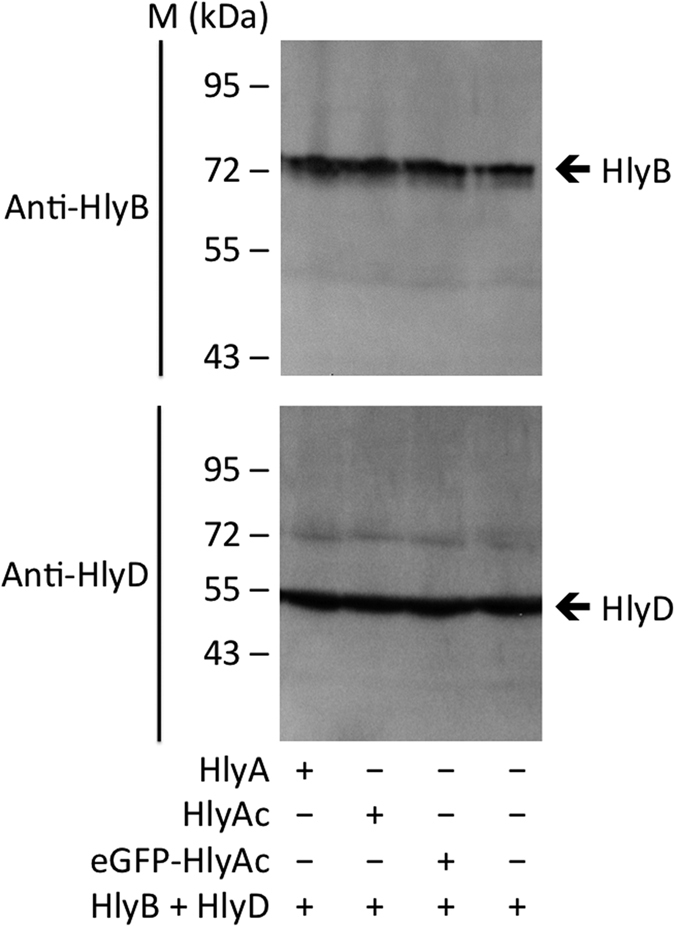
HlyB and HlyD expression levels. Western blot analysis of *E. coli* cells demonstrated that the expression levels of HlyB and HlyD were equal for cells expressing and/or secreting either eGFP-HlyAc, HlyA or HlyAc.

Using the results of experiments with different durations of secretion, an average value of at least 16.0 ± 1.3 aa T1SS^−1^s^−1^ was determined for HlyA. Following the same line of experiments (for the corresponding gels see Supplementary Fig. 3 for HlyA and Supplementary Fig. 4 for HlyAc), the secretion rate of HlyAc was calculated to at least 16.6 ± 1.4 aa T1SS^−1^s^−1^. Western blot analysis using the polyclonal antibodies against HlyB and HlyD confirmed that the expression levels of both proteins were similar in cells expressing the T1SS and were not effected by the co-expression of the substrates (Fig. 2).

The results described above demonstrated that the secretion rates of HlyA and HlyAc were identical within experimental error. However, the absolute number of secreted HlyA molecules was 4 to 5 fold lower compared to HlyAc. This simply reflects the difference in number of amino acids, 1024 aa in the case of HlyA and 218 aa in the case of HlyAc. Consequently, the number of secreted molecules of HlyAc is accordingly higher compared to HlyA.

### ATP hydrolysis is necessary for early step of secretion

Binding and/or hydrolysis of ATP by the ABC transporter HlyB provides the necessary energy for substrate translocation through the T1SS. Previous studies have demonstrated that substrate secretion is abolished in the absence of ATP hydrolysis but did not affect complex assembly^21^. Nevertheless, since the exact step for which ATPase activity is required remained elusive, the question arises, whether an early step such as entering the translocation pathway or a late step of secretion such as release from the cell envelop or re-setting of the T1SS requires an energetic input.

To analyze the importance of ATP hydrolysis during substrate secretion, the H662A mutant of HlyB, which is deficient in ATP hydrolysis, was used. This mutant is able to bind ATP, dimerization of the NBDs but does not catalyze ATP hydrolysis^27^,^29^. In combination with the concept of a stalled T1SS complex described above, the H662A mutant might help to clarify which step of substrate translocation is coupled to ATP hydrolysis.

**Figure 3:**
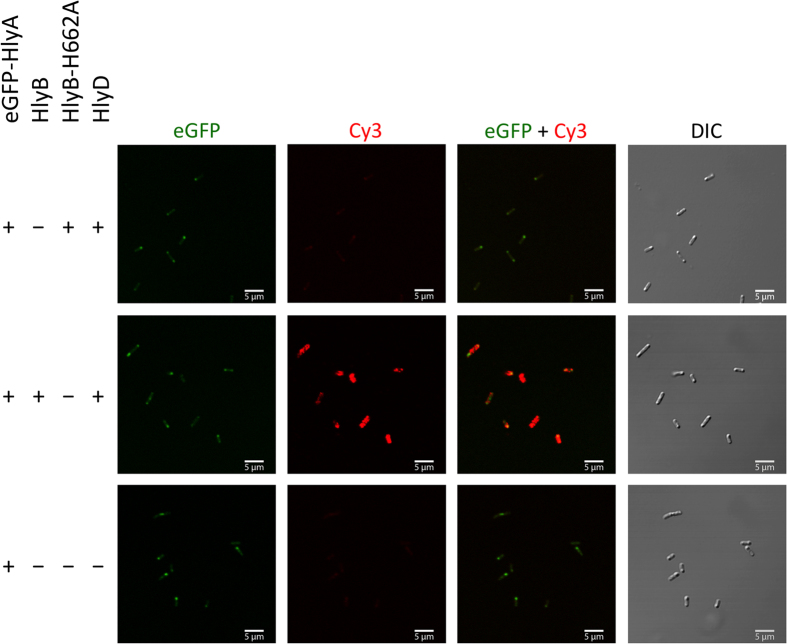
Detection of the surface exposed HlyA fragment of eGFP-HlyA by confocal laser scanning microscopy. *E. coli* cells expressing eGFP-HlyA, HlyD, HlyB or HlyB-H662A in different combinations of the proteins. Shown is the eGFP fluorescence (left panel) of the fusion proteins, the HlyA mediated Cy3 fluorescence at the cell surface (second left panel), merged images of eGFP and Cy3 fluorescence (second right panel) and differential interference contrast (DIC) images of the cells (right panel). The different combinations of proteins employed are indicated to the left.

*E. coli* cells expressing a T1SS harboring the HlyB H662A mutant (Fig. 3, first row) were analyzed by confocal laser scanning microscopy (CLSM). Homogenously distributed cytosolic eGFP fluorescence was observed, which sometimes accumulated at the cell poles. However, no Cy3 fluorescence was detected at the cell surface indicating that the C-terminal part of HlyA did not reach the extracellular space. As a control, *E. coli* cells expressing eGFP-HlyA but not HlyB and HlyD were also analyzed (Fig. 3, third row). Here, similar, cytosolic eGFP fluorescence was observed, but again no Cy3 fluorescence was detected (for quantification of the observed eGFP and Cy3 see Supplementary Fig. 5).

In summary, these results demonstrated that the eGFP-HlyA fusion protein was only presented at the cell surface if HlyB was capable of binding and hydrolyzing ATP. This strongly suggests that ATP hydrolysis is essential for an early event in the translocation process.

### The role Ca^2+^ on the rate of secretion

The requirement of an unfolded substrate during translocation and the directionality of secretion, i.e. the C-terminal part is presented at the cell surface first, is in line with the hypothesis that binding of Ca^2+^ to the GG repeats and the subsequent folding represents the driving force of substrate secretion by T1SS^10^. The affinity of the GG repeats of HlyA and the concentration of Ca^2+^ in the cytosol of *E. coli* and the extracellular medium support this scenario, in which Ca^2+^ induced protein folding creates an inherent driving force that “pulls” the protein out of the translocator^10^,^11^.

We addressed the question of a ‘Ca^2+^-triggered pulling mechanism’ by varying the Ca^2+^ concentration in the extracellular medium. If such a ‘pulling’ mechanism indeed provides the inherent driving force for substrate transport, the secretion rate has to depend on the external Ca^2+^ concentration. Below the K_D_ of the GG repeats for Ca^2+^ (100 μM^16^), a clear drop in the rate should be apparent, while the rate should remain constant at Ca^2+^ concentrations exceeding the K_D_.

HlyA and HlyAc are unstable in the absence of Ca^2+^, or at concentration below the K_D_ of the GG repeats and an aggregation of the secreted substrates was observed. However, extracellular aggregation appeared only if HlyA and HlyAc reached a critical concentration, typically after three hours resulting in visible aggregation at the rim of the incubation flasks. Therefore, the time of the secretion experiments was restricted to two hours.

**Figure 4:**
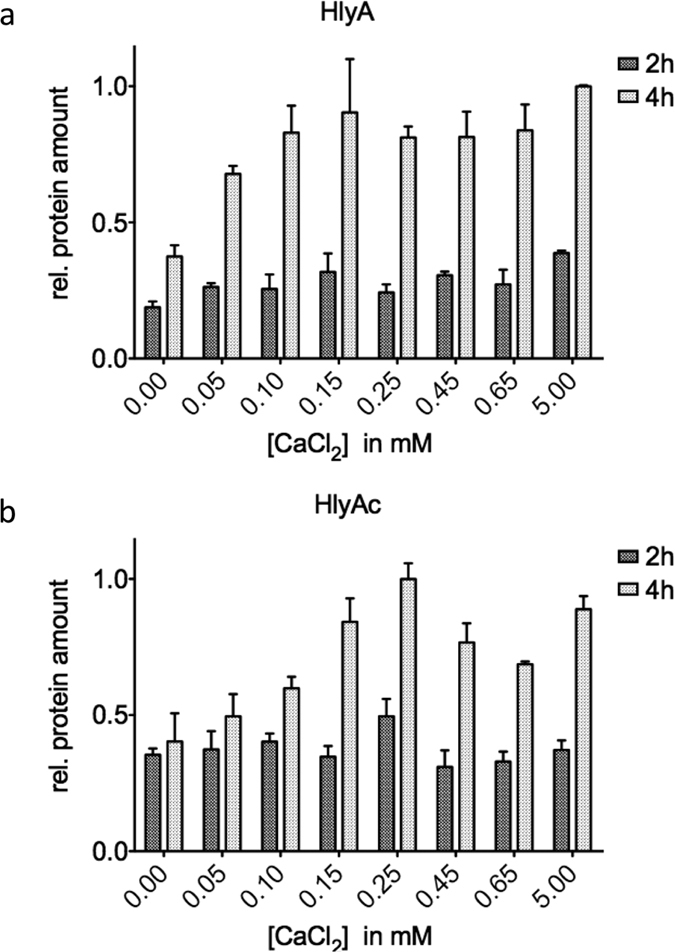
Relative amount of secreted HlyA and HlyAc in the presence of different extracellular Ca^2+^ concentrations. The relative amount of secreted HlyA (**a**) and HlyAc (**b**) in the presence of different extracellular Ca^2+^ concentration after 2 h (dark grey bars) and 4 h (light grey bars) is summarized. Values are normalized to the highest mean value of secreted HlyA (**a**) or HlyAc (**b**) after 4 h of secretion. Error bars represent the standard deviation of at least three biological replicates.

In all of the secretion experiments addressing the role of the concentration of extracellular Ca^2+^, the Ca^2+^ concentration was adjusted either by addition of external Ca^2+^ or EGTA and verified by atomic absorption spectroscopy (for further details see Materials & Methods). The amount of both substrates and HlyB/HlyD remained at the same level during the first two hours of secretion, independently of the external Ca^2+^ concentration (Fig. 4 dark grey bars and Supplementary Fig. 8).

**Figure 5:**
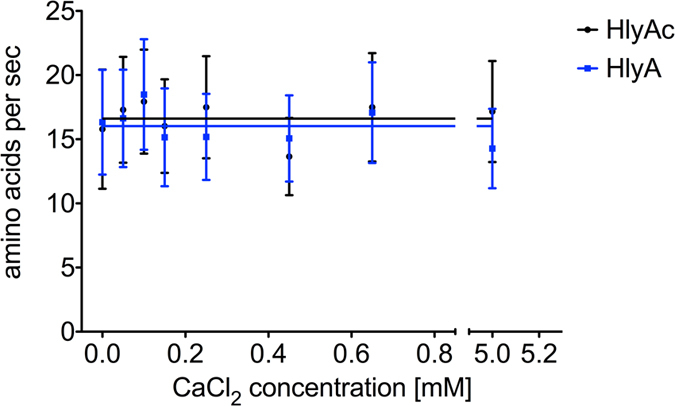
The secretion rate of HlyA and HlyAc is independent of the extracellular Ca^2+^ concentration. Determined secretion rates of HlyA (blue cubes) and HlyAc (black dots) in the presence of different Ca^2+^ concentration. Shown are the mean secretion rates of triplicate secretion experiments as amino acids (aa) T1SS^−1^ s^−1^. Error bars represent the standard deviation of at least three biological replicates.

The secretion rates for HlyA and HlyAc at different extracellular Ca^2+^ concentrations are summarized in Supplementary Tables 2 and 3. The results highlight that the secretion rate of HlyA remained constant between 14.3 ± 3.1 and 18.5 ± 4.3 aa T1SS^−1^ s^−1^, respectively, while values between 15.8 ± 4.1 aa T1SS^−1^ s^−1^ and 17.9 ± 4.1 aa T1SS^−1^ s^−1^ were determined for HlyAc. These results demonstrated that therefore a Ca^2+^ concentration between 0 and 5 mM did not alter the secretion rate (Fig. 5).

Ca^2+^ ions are essential for the stability and correct folding of HlyA and HlyAc, respectively. However, our experiments suggest that Ca^2+^ does not provide an inherent driving force for secretion of HlyA and does therefore not impose a “pulling” mechanism during the secretion process. One might speculate that as an alternative to the absolute requirement of ATP hydrolysis, an entropic driving force acts in T1SS as was proposed for protein synthesis at the ribosome^30^. Here one could envision that confinement of the tunnel-channel of a T1SS translocon, entropically favors formation of secondary structure elements that prevent back-sliding and thereby impose a force that pulls HlyA and some other substrates out of the translocon. However, the question raised is whether the conformation and/or structure of HlyA that is secreted in the absence of Ca^2+^ is similar to the one adopted in the presence of Ca^2+^. Therefore, we measured the intrinsic Trp fluorescence of HlyA in the presence or absence of 2 mM Ca^2+^ at 331 nm and 350 nm, respectively^16^. For HlyA secreted in the presence of 2 mM Ca^2+^, we obtained a ratio of the intrinsic Trp fluorescence at 350 nm to 331 nm of 1.44 ± 0.05 indicating a properly folded protein^16^. In contrast, the ratio 350 nm over 331 nm of HlyA secreted in the absence of Ca^2+^ was 0.63 ± 0.03 suggesting that HlyA adopted a different conformation under these conditions. To assess this difference further, we secreted acylated HlyA (the lytically active form of HlyA) in the presence and absence (2 mM) Ca^2+^ and placed 10 μl of supernatant on Columbia blood agar plates. After 2 h of incubation at 37 °C, clear halo formation was visible for acylated HlyA secreted in the presence of 2 mM Ca^2+^ (Supplementary Fig. 6). This clearly showed that acylated HlyA was properly folded. In clear contrast, no halo formation was detected in the case of acylated HlyA secreted in the absence of Ca^2+^ suggesting that acylated HlyA adopted a non-lytic conformation under this condition (Supplementary Fig. 6).

## Discussion

The concept of a stalled HlyA T1SS by an HlyAc fusion with rapidly folding eGFP was recently used to determine the orientation of HlyA during secretion^20^. We used this approach to quantify the number of active T1SS per cell. Here, we assume that on average only one HlyA antibody and one Cy3-labeled secondary antibody binds to a single, surface-exposed HlyAc. Employment of a second, commercially available and Cy3-labeled antibody supported this assumption. Furthermore, we also determined the total number of HlyB molecules that are present in the inner membrane of *E. coli* cells by Western blot analysis. Assuming that HlyB like all ABC transporters analyzed so far forms a dimer as the functional unit^31^, this method revealed the absolute number of HlyB molecules, thus imposing an upper limit to the amount of T1SS present per cell. This value supported our fluorometric-determined value of approximately 4500 active T1SS per cell (Supplementary Table 1). This allowed us to determine the secretion rate of T1SS *in vivo* for the first time. The velocity of secretion of HlyA was determined to be 16.0 ± 1.3 aa T1SS^−1^ s^−1^, while HlyAc was secreted at a velocity of 16.6 ± 1.4 aa T1SS^−1^s^−1^. This demonstrates that the secretion rate per transporter and per cell is independent of the size of the substrate.

Previous studies highlighted the importance of ATP hydrolysis for protein translocation in the HlyA T1SS^21^. HlyA secretion is completely abolished by using an HlyB mutant protein deficient in ATP hydrolysis. Nevertheless, cross-linking experiments in the same study confirmed that the translocator still assembled even in the absence of ATP consumption. However, the particular step for which ATP hydrolysis is required was not identified^21^. Our results additionally demonstrate that ATP hydrolysis is essential for the substrate to reach the cell envelope. This leads to two fundamentally different models of how the HlyA T1SS operates. In the first model, continuous ATP consumption is necessary for energizing the transport process. In this case, a defined number of ATP molecules are consumed per transported amino acid(s). Such an iterative mechanism was suggested for SecA-dependent protein translocation^25^,^26^. Here, a rate of 152–228 amino acids per sec and transporter can be calculated based on experimental data obtained for SecA *in vivo*^24^. This process is faster than the secretion rate of at least 16.6 ± 1.4 aa T1SS^−1^ s^−1^ determined for the HlyA T1SS. However, the Sec pathway is one of the most essential nanomachineries in Gram-negative bacteria and responsible for the integration of most transmembrane proteins and the transport for many secreted proteins. In the second model, ATP hydrolysis could act as an effector similar to ATP-gated channels. Here, ATP hydrolysis would initiate channel opening and allow translocation of the substrate through the HlyA T1SS pore. In such a scenario, ATP consumption as the driving force for discrete amounts of transported amino acids per ATP consumed is highly unlikely. T1SS substrates can have a size of more than 8000 aa^2^,^4^ and hydrolysis of only one or two ATP molecules per translocated substrate seems very unlikely.

Other possibilities that can energize the transport process are the proton motive force, which plays an important role in T3SS^32^ and at an early stage of HlyA secretion^22^, or diffusion along a concentration or an electrostatic gradient. The proposed Ca^2+^-dependent “pulling” mechanism for HlyA^14^,^18^ as the inherent driving force is unlikely due to the result of our experiments.

The presence of GG repeats just upstream of the C-terminal secretion sequence is common to a large number of substrates of T1SS^2^. In the case of HlyA, the GG repeats bind Ca^2+^ with an affinity of approximately 100 μM^13^,^14^,^15^,^16^ and trigger folding of the protein at the extracellular surface due to the presence of high Ca^2+^ concentration in the extracellular medium^17^,^33^,^34^. Extracellular protein folding could involve an inherent driving force, which ‘pulls’ the protein out of the translocon. Only recently, Bumba *et al*. reported that Ca^2+^ indeed contributed to such a pulling force for the adenylate cyclase toxin CyaA from *Bordetella pertussis*^19^. In an elegant set of experiments, it was demonstrated that CyaA, a 1706 amino acid member of the RTX protein family, requires a C-terminal cap and Ca^2+^ ions in the extracellular media to ensure efficient secretion. In this scenario, binding of Ca^2+^ induces a Brownian ratchet mechanism that accelerates secretion. Our results are in contrast to these data^19^ and other proposals^14^,^18^. After two hours of secretion, the secretion rate did not show any dependence on the extracellular Ca^2+^ concentration. Even in the absence of Ca^2+^ no significant deviation was observed. These results indicate that the proposed “pulling” mechanism is not operational in HlyA and that Ca^2+^ binding to the GG repeats is solely important to ensure proper folding and stability of the substrate. This assumption is supported by our data using intrinsic Trp fluorescence and data on the activity of acylated HlyA (Supplementary Fig. 6). Here, the value of intrinsic Trp fluorescence of 0.63 ± 0.03 (ratio at 331 nm over 350) of HlyA secreted in the absence of Ca^2+^ is identical within experimental error to HlyA unfolded in 8 M urea (0.062 ± 0.05)^16^. Thus the secretion rate of HlyA in the absence of Ca^2+^ is not influenced, although the protein adopts a non-active conformation in the absence of Ca^2+^.

Furthermore, one has to stress that HlyA (1024 amino acids) harbors 6 GG repeats, while CyaA (1706 amino acids) harbors 17 GG repeats^9^. Thus, the increased size of CyaA and the higher numbers of GG repeats might regive an additional pulling force that is necessary for efficient secretion of this particular type 1 protein.

In summary, our results provided for the first time a quantitative measurement of the *in vivo* secretion rate of a T1SS. Furthermore, the proposed “pulling” mechanism of Ca^2+^ binding to the GG repeats as driving force could be excluded. On the other hand, ATP hydrolysis is essential for the early steps of substrate translocation.

## Methods

### Bacterial strains and plasmids

*E. coli* DH5α cells were used for all cloning procedures. The pK184 plasmid (Supplementary Fig. 7) was used for HlyB and HlyD expression under the control of a P_*lac*_promotor, inducible with IPTG (isopropyl-β-D-1-thiogalactopyranoside)^12^. All plasmids and oligonucleotides used in this study are summarized in Supplementary Tables 4 and 5. Plasmids pSOI-eGFP-HlyAc and pSOI-eGFP-HlyA, respectively, were used for eGFP-HlyAc or eGFP-HlyA expression under the control of a P_BAD_ promoter^20^. Plasmids pSU-*hlyA* and pSU-*hlyA1* were used for HlyA and HlyAc expression, respectively, under the control of a P_*lac*_promotor, inducible with IPTG (isopropyl-β-D-1-thiogalacto- pyranoside)^16^,^35^. The HlyB-H662A mutant was expressed using a variant of the pK184 plasmid (pK184-HlyB-H662A-HlyD).

### Cell cultivation and protein expression for confocal laser scanning microscopy

Chemically competent *E. coli* BL21 (DE3) cells were transformed with or without pK184-HlyBD or pK184-HlyB-H662A-HlyD and pSOI-eGFP-HlyAc or pSOI-eGFP-HlyA, respectively and grown at 37 °C on LB agar plates supplemented with 100 μg mL^−1^ ampicillin and/or 30 μg mL^−1^ kanamycin. *E. coli* BL21 (DE3) cells were prepared and induced as previously described^20^.

### Confocal laser scanning microscopy and image processing

Microscopy and image processing were carried out as previously described^20^. We present here single confocal planes that do not contain maximum intensity projections composed of z scans.

### Fluorescence spectrometry of immunofluorescence labeled formaldehyde treated cells

Immunofluorescence labeled and formaldehyde treated *E. coli* BL21 (DE3) cells expressing eGFP-HlyAc with and without HlyB/HlyD were adjusted to an OD_600_ of 1.0 and were analyzed by a fluorescence spectrometry (Jobin-Ivon Horiba Fluorolog-3) to quantify the amount of T1SS. All measurements were performed at 25 °C in a 50 μL cuvette. Excitation was performed at 547 nm and fluorescence emission was monitored at 563 nm with slit widths of 5 nm each. Fluorescence was recorded for 0.5 s. An identical experiment was carried out with PBS buffer and different concentration of free Cy3 fluorophore-linked antibody (0.5 pM–1.5 μM).To validate the results of the Cy3-labeled antibody, we used another, commercially available (Molecular Probes) Cy3-labeled antibody. This antibody was used as described above for the first Cy3-labeled antibody.

### Secretion experiments with HlyA and HlyAc in the presence of different CaCl_2_ concentrations

Chemically competent *E. coli* BL21 (DE3) cells were transformed with pK184-HlyBD and pSU-*hlyA* or pSU-*hlyA1* and grown on LB agar plates supplemented with 100 μg mL^−1^ ampicillin and 30 μg mL^−1^ kanamycin. Ca^2+^ concentration of 2YT medium was determined by atomic absorption spectroscopy.

Overnight cultures of single colonies were used to inoculate 25 mL 2YT medium supplemented with 100 μg mL^−1^ ampicillin and 30 μg mL^−1^ kanamycin at an OD_600_ of 0.1. Cultures were grown at 37 °C and 180 rpm. The expression of HlyA, HlyAc, HlyB and HlyD was induced by addition of 1 mM IPTG at an OD_600_ of 0.6–0.8. EGTA, respectively CaCl_2_, were added at this point to adjust the final Ca^2+^ concentration in the culture medium. Cells were grown for 4 h at 180 rpm and 37 °C. A 1 mL aliquot was taken each hour during cell growth and centrifuged for 5 min at 14 000 g, 4 °C. The supernatant was diluted one to eight and analyzed by SDS PAGE (Supplementary Figs 3 and 4).

A dilution series of purified HlyA of known concentration ranging from 40 μg mL^−1^ to 625 ng mL^−1^ or HlyAc of known concentrations ranging from 50 μg mL^−1^ to 758 ng mL^−1^ were loaded on a SDS-PAGE. Gels were stained using Coomassie Brilliant Blue (CBB). The expression levels of HlyB and HlyD as well as the expression of intracellular amount of HlyA and HlyAc were determined by Western blots (Supplementary Fig. 8) using polyclonal antibodies against HlyA, HlyB and HlyD, respectively, in combination with an horseradish peroxidase (HRP)-conjugated, secondary antibody using the ECL advance kit (GE Healthcare).

### Data processing of the secretion experiments

ImageJ^36^ was used for processing and determination of the band intensities of the proteins of interest on SDS-PAGE gels. The intensity of the band of purified HlyA and HlyAc signals of the dilution series was used to determine the concentration of the secreted HlyA and HlyAc, respectively. The slop of the plotted HlyA or HlyAc amount represents the amount of secreted HlyA or HlyAc per time. This amount can be divided by the growth factor of the cells in their exponential growing phase. The quotient represents the amount of secreted HlyA or HlyAc per time and cell and can be transformed by the amount of T1SS per cell and the number of amino acids of HlyA or HlyAc to finally yield the secretion rate in number of amino acids per sec and T1SS.

### Determination of the amount of T1SS by HlyB Western blot analysis

Cells used in the secretion experiments of HlyA and HlyAc, respectively, were used to determine the number of HlyB molecules per cell. For this purpose, we used cells after 2 h of secretion. Here, cells of the 1 mL aliquot were collected by centrifugation (1 min at 14,000 × g, 4 °C) and the cell pellet was re-suspended in water to obtain 0.5 × 10^7^ cells/μl. 16 μl of this samples and 4 μl SDS PAGE buffer was loaded on a SDS-PAGE for subsequent analysis by Western blots analysis as described above. A concentration of purified HlyB ranging from 9 μg mL^−1^ to 144 ng mL^−1^ was analyzed by Western blot and signal intensities of the HlyB signals were determined using the program ImageJ^36^. The intensity of purified HlyB signals of the dilution series were used to determine the concentration of expressed HlyB per cell. This number was divided by the number of cells. The calculation resulted in the amount of HlyB per cell. Since the functional unit of HlyB is a dimer, division by two resulted in the total number of T1SS per cell.

### Purification of HlyA, HlyAc and HlyB for regression analysis

Purification of HlyA and HlyAc was carried out as previously described^16^,^35^. HlyB was purified as described^29^. The concentration of the purified protein was determined spectrophotometrically (Nanodrop-1000, Thermo Scientific) using the calculated (web.expasy.org/protparam) extinction coefficient at 280 nm.

### Functional analysis of HlyA and acylated HlyA

HlyA and acylated HlyA were secreted in the absence or presence of Ca^2+^ (2 mM) and purified as described^16^. Intrinsic Trp fluorescence of both proteins was measured at 350 nm and 330 nm, respectively. The excitation wavelength was set to 290 nm, slit width to 5 nm and fluorescence was recorded for 0.5 s. The ratio of both values was used as an indicator of the folding state as described^16^. The ratio of the intrinsic Trp fluorescence at 350 nm over 331 of HlyA unfolded in 8 M urea is 0.62 ± 0.05^16^.

### Halo assay of the hemolytic activity of acylated HlyA

Acylated HlyA was secreted in the absence or presence of Ca^2+^ (2 mM). Therefore, 25 mL 2YT medium supplemented with 100 μg mL^−1^ ampicillin and 30 μg mL^−1^ kanamycin were inoculated with acylated HlyA secreting cells^16^ at an OD_600_ of 0.1. Cultures were grown at 37 °C and 180 rpm. The expression of acylated HlyA, HlyB and HlyD was induced by addition of 1 mM IPTG at an OD_600_ of 0.6–0.8. EGTA, respectively CaCl_2_, were added at this point to adjust the final Ca^2+^ concentration in the culture medium. Cells were grown for 2 h at 180 rpm and 37 °C. After 2 h of secretion, cells were separated by centrifugation (5 min at 14 000 g and 4 °C) and 10 μl of supernatant was put on the Columbia blood agar plates. Plates were incubated for 2 h at 37 °C and halo formation was observed.

## Additional Information

**How to cite this article**: Lenders, M. H. H. *et al*. *In vivo* quantification of the secretion rates of the hemolysin A Type I secretion system. *Sci. Rep.* **6**, 33275; doi: 10.1038/srep33275 (2016).

## Supplementary Material

Supplementary Information
