# Supplementary material for: In vivo quantification of the secretion rates of the hemolysin A Type I secretion system
Source: Sci Rep. 2016 Sep 12;6:33275. doi: 10.1038/srep33275 (PMC5018854; doi:10.1038/srep33275)
Supplement: Supplementary Information [file srep33275-s1.doc]

Supplementary

***In vivo* quantification of the secretion rates of the
hemolysin A Type I secretion system**

Michael H. H. Lenders^1^, Tobias Beer^1^, Sander H. J. Smits^1^ and Lutz Schmitt^1^*

^1^Institute of Biochemistry, Heinrich-Heine-Universitaet, 40225 Duesseldorf, Germany

*To whom correspondence should be addressed:

Lutz.Schmitt@hhu.de

Tel. +49 211 81-10773

Fax +49 211 81-15310

Universitaetsstraße 1

40225 Duesseldorf

Germany

Figures legends

Supplementary Figure 1

Regression analysis of Cy3 fluorescence of the two (a, b) Cy3-labeled secondary antibodies in solution. The insets show the data at low antibody concentrations.

Supplementary Figure 2

Integrated area of Western blot signals of purified HlyB at different concentrations of HlyB.

Supplementary Figure 3

Secretion level of HlyA in the presence of different extracellular Ca^2+^ concentration. SDS-PAGE analysis of the HlyA secretion level in the culture supernatant over a four-hour time period. (a) SDS-PAGE of purified HlyA at different concentrations. The concentrations are indicated above the gel. (b) Evaluation of the band intensities of HlyA of the SDS PAGE analysis at 5mM Ca^2+^ (black line) and evaluation of the number of cells (blue line) present during the duration of the secretion experiment. (c) SDS-PAGEs show the HlyA secretion levels after every hour. Different extracellular Ca^2+^ concentrations are indicated above the gels. Bar diagrams below the SGS-PAGEs show the relative intensity of the SDS-PAGE bands. Bands are normalized on the highest mean value of secreted HlyA at a particular Ca^2+^ concentration after 4 h secretion time. Error bars represent the standard deviation of at least three biological replicates.

Supplementary Figure 4

Secretion level of HlyAc in the presence of different extracellular Ca^2+^ concentration. SDS-PAGE analysis of the HlyAc secretion level in the culture supernatant over a four-hour time period. (a) SDS-PAGE of purified HlyAc at different concentrations. The concentrations are indicated above the gel. (b) Evaluation of the band intensities of HlyAc of the SDS PAGE analysis at 5mM Ca^2+^ (black line) and evaluation of the number of cells (blue line) present during the duration of the secretion experiment. (c) SDS-PAGEs show the HlyAc secretion level after every hour. Different extracellular Ca^2+^ concentrations are indicated above the gels. Bar diagrams below the SGS-PAGEs show the relative intensity of the SDS-PAGE bands. Bands are normalized on the highest mean value of secreted HlyAc at a particular Ca^2+^ concentration after 4 h secretion time. Error bars represent the standard deviation of at least three biological replicates.

Supplementary Figure 5

(a) Relative cell fluorescence of eGFP. All values were normalized to the eGFP fluorescence of the eGFP-HlyA fusion protein (error bars represent the standard error of the mean of at least three independent experiments). (b) Relative fluorescence of a the secondary Cy3-coupled antibody. All values were normalized to the Cy3 fluorescence of the eGFP-HlyA fusion protein (error bars represent the standard error of the mean of at least three independent experiments). The different combinations of proteins are indicated below the bar plots.

Supplementary Figure 6

Halo assay of the hemolytic activity of acylated HlyA on Columbia blood agar plates. Acylated HlyA was secreted in the absence (left panel) and 2 mM Ca2+ (right panel). After 2 h of secretion, cells were separated by centrifugation, 10 µl of supernatant was put on the agar plats and plates were incubated for 2h at 37 °C (for further details see Materials and Methods).

Supplementary Figure 7

Plasmid map pK184-HlyB-H662A-HlyD

Supplementary Figure 8

Western blot analysis of cell content after 2 h of secretion at different Ca^2+^ concentrations. HlyA, HlyAc, HlyB and HlyD, respectively, are only detected if their expression was induced. Their total amount of the proteins remains constant during the time period of the secretion experiments performed in the presence of different Ca^2+^concentration.

Tables

Supplementary Table 1

Mean value of transporter

|  | Transporter |
| --- | --- |
| by fluorescence (antibody 1)  by fluorescence (antibody 2)  by fluorescence (average) | 4509 ± 1061  4554 ± 1616  4532 ± 966 |
| by western blot | 5355 ± 483 |

Supplementary Table 2

Calculation of HlyA transport rates at different Ca^2+^concentration

| Condition | # | Secreted HlyA after  2h 4h  [pmol] [pmol] | | Secreted HlyA  [mol min^-1^] | Cell growth  [cells min^-1^] | Rate by fluorescence  [aa T1SS^-1^ s^-1^] |
| --- | --- | --- | --- | --- | --- | --- |
| 0.00mM CaCl_2_ | 1 | 5.2 | 9.9 | 7.46 ± 0.72 | 1.04 ± 0.09 | 16.3 ± 4.1 |
|  | 2 | 4.4 | 8.0 |  |  |  |
| 0.05mM CaCl_2_ | 1 | 6.8 | 17 | 8.93 ± 0.22 | 1.22 ± 0.09 | 16.6 ± 3.8 |
|  | 2 | 6.4 | 17 |  |  |  |
|  | 3 | 6.1 | 16 |  |  |  |
| 0.10mM CaCl_2_ | 1 | 5.5 | 18 | 10.9 ± 0.80 | 1.33 ± 0.08 | 18.5 ± 4.3 |
|  | 2 | 5.5 | 21 |  |  |  |
| 0.15mM CaCl_2_ | 1 | 9.6 | 28 | 12.6 ± 1.42 | 1.89 ± 0.14 | 15.2 ± 3.8 |
|  | 2 | 7.5 | 20 |  |  |  |
| 0.25mM CaCl_2_ | 1 | 6.3 | 20 | 10.3 ± 0.51 | 1.53 ± 0.04 | 15.2 ±3.4 |
|  | 2 | 5.1 | 21 |  |  |  |
|  | 3 | 6.5 | 19 |  |  |  |
| 0.45mM CaCl_2_ | 1 | 7.9 | 21 | 10.4 ± 0.54 | 1.57 ± 0.06 | 15.1 ±3.4 |
|  | 2 | 7.2 | 22 |  |  |  |
|  | 3 | 7.4 | 17 |  |  |  |
| 0.65mM CaCl_2_ | 1 | 5.8 | 18 | 10.8 ± 0.69 | 1.44 ± 0.08 | 17.1 ± 3.9 |
|  | 2 | 6.1 | 21 |  |  |  |
|  | 3 | 8.2 | 23 |  |  |  |
| 5.00mM CaCl_2_ | 1 | 9.6 | 25 | 12.8 ± 0.16 | 2.03 ± 0.08 | 14.3 ± 3.1 |
|  | 2 | 9.7 | 24 |  |  |  |
|  | 3 | 9.3 | 25 |  |  |  |

Supplementary Table 3

Calculation of HlyAc transport rates at different Ca^2+^concentration

| Condition | # | Secreted HlyA after  2h 4h  [pmol] [pmol] | | Secreted HlyA  [mol min^-1^] | Cell growth  [cells min^-1^] | Rate by fluorescence  [aa T1SS^-1^ s^-1^] |
| --- | --- | --- | --- | --- | --- | --- |
| 0.00mM CaCl_2_ | 1 | 54 | 70 | 38.5 ± 6.85 | 1.18 ± 0.12 | 15.8 ± 4.6 |
|  | 2 | 47 | 44 |  |  |  |
|  | 3 | 37 | 45 |  |  |  |
| 0.05mM CaCl_2_ | 1 | 50 | 65 | 52.2 ± 5.12 | 1.46 ± 0.06 | 17.3 ± 4.1 |
|  | 2 | 40 | 56 |  |  |  |
|  | 3 | 36 | 46 |  |  |  |
| 0.10mM CaCl_2_ | 1 | 44 | 69 | 53.1 ± 3.38 | 1.44 ± 0.06 | 17.9 ± 4.1 |
|  | 2 | 43 | 62 |  |  |  |
|  | 3 | 49 | 71 |  |  |  |
| 0.15mM CaCl_2_ | 1 | 39 | 55 | 48.4 ± 3.24 | 1.46 ± 0.06 | 16.0 ± 3.6 |
|  | 2 | 43 | 100 |  |  |  |
|  | 3 | 34 | 88 |  |  |  |
| 0.25mM CaCl_2_ | 1 | 47 | 110 | 53.9 ± 2.95 | 1.49 ± 0.09 | 17.5 ± 4.0 |
|  | 2 | 60 | 120 |  |  |  |
|  | 3 | 60 | 110 |  |  |  |
| 0.45mM CaCl_2_ | 1 | 41 | 95 | 42.6 ± 1.96 | 1.51 ± 0.05 | 13.7 ± 3.0 |
|  | 2 | 36 | 84 |  |  |  |
|  | 3 | 27 | 80 |  |  |  |
| 0.65mM CaCl_2_ | 1 | 54 | 130 | 54.9 ± 5.58 | 1.52 ± 0.08 | 17.5 ± 4.2 |
|  | 2 | 38 | 100 |  |  |  |
| 5.00mM CaCl_2_ | 1 | 44 | 110 | 50.4 ± 1.71 | 1.42 ± 0.11 | 17.2 ± 3.9 |
|  | 2 | 37 | 96 |  |  |  |
|  | 3 | 44 | 98 |  |  |  |

Supplementary Table 4

Primers used in this study

| Name | Sequence |
| --- | --- |
| H662A_5for_B-NBD | 5’-CGGTTATAATCATTGCTGCGCGTCTGTCTACAGTAA-3’ |
| H662A_3rev_B-NBD | 5’-TTACTGTAGACAGACGCGCAGCAATGATTATAACCG-3’ |

Supplementary Table 5

Plasmids used in this study

| Name | Description | Reference |
| --- | --- | --- |
| pK184-HlyB  pK184-HlyB-H662A-HlyD  pSU-*hlyA*  pSU-*hlyA1*  pSOI-eGFP-HlyAc  pSOI-eGFP-HlyA | Plasmid encoding *hlyB* and *hlyD*  Plasmid pK184-HlyB with a base pair substitution to generate *hlyB-H662A* *via* site-directed mutagenesis; encodes for *hlyB-H662A* and *hlyD*  Plasmid encoding *hlyA*  Plasmid encoding *hlyAc*  Plasmid encoding *eGFP-hlyAc*  Plasmid encoding *eGFP-hlyA* | [^1^](#_ENREF_1)  This study  [*^2^*](#_ENREF_2)  [^3^](#_ENREF_3)  [^4^](#_ENREF_4)  [^4^](#_ENREF_4) |

References

1. Bakkes PJ, Jenewein S, Smits SH, Holland IB, Schmitt L. The rate of folding dictates substrate secretion by the Escherichia coli hemolysin type 1 secretion system. *J Biol Chem* **285**, 40573-40580 (2010).

2. Thomas S, Bakkes PJ, Smits SH, Schmitt L. Equilibrium folding of pro-HlyA from Escherichia coli reveals a stable calcium ion dependent folding intermediate. *Biochim Biophys Acta* **1844**, 1500-1510 (2014).

3. Lecher J, Schwarz CK, Stoldt M, Smits SH, Willbold D, Schmitt L. An RTX transporter tethers its unfolded substrate during secretion via a unique N-terminal domain. *Structure* **20**, 1778-1787 (2012).

4. Lenders MH, Weidtkamp-Peters S, Kleinschrodt D, Jaeger KE, Smits SH, Schmitt L. Directionality of substrate translocation of the hemolysin A Type I secretion system. *Sci Rep* **5**, 12470 (2015).


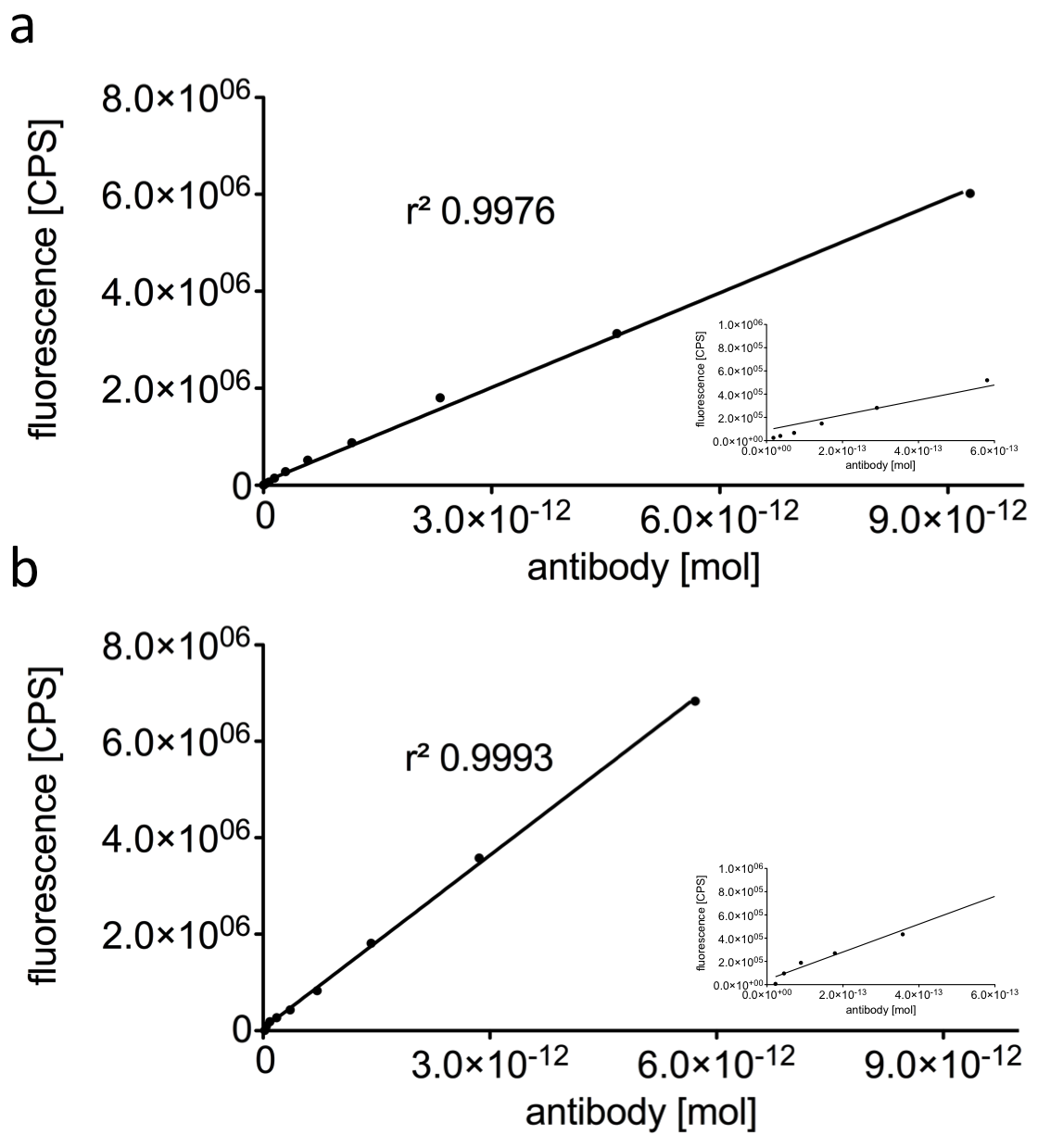


Supplementary Fig. 1


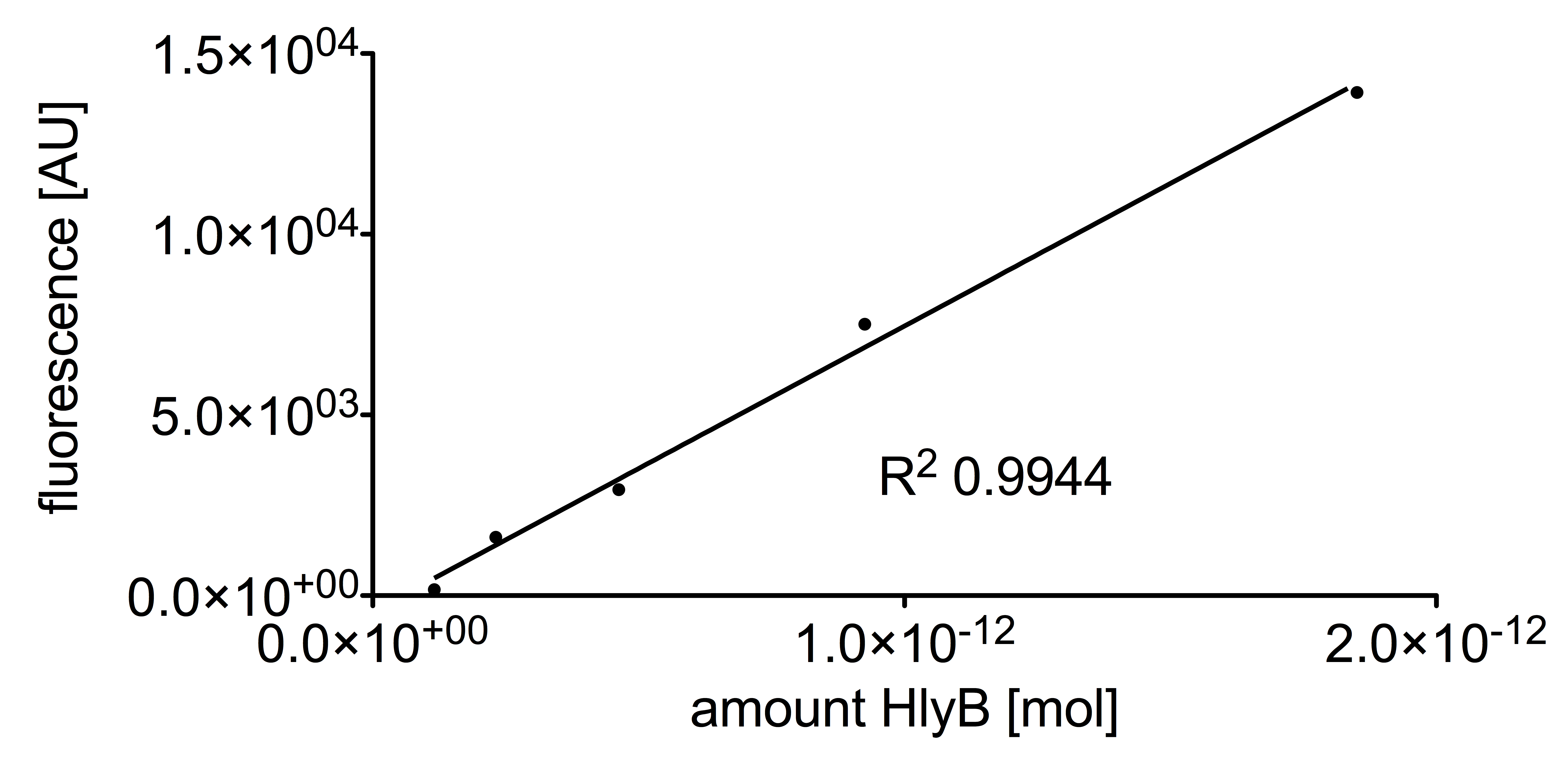


Supplementary Fig. 2


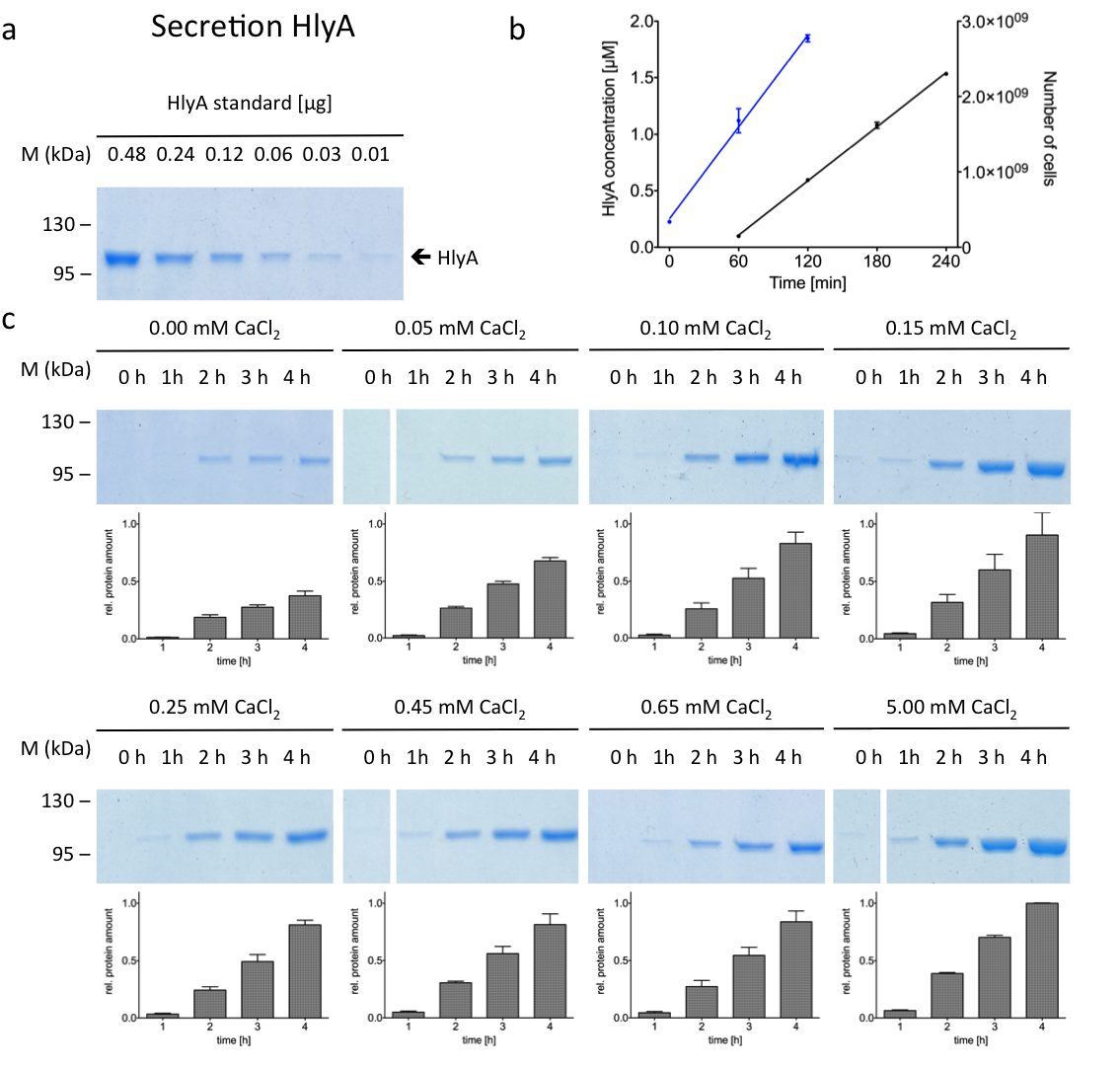


Supplementary Fig. 3


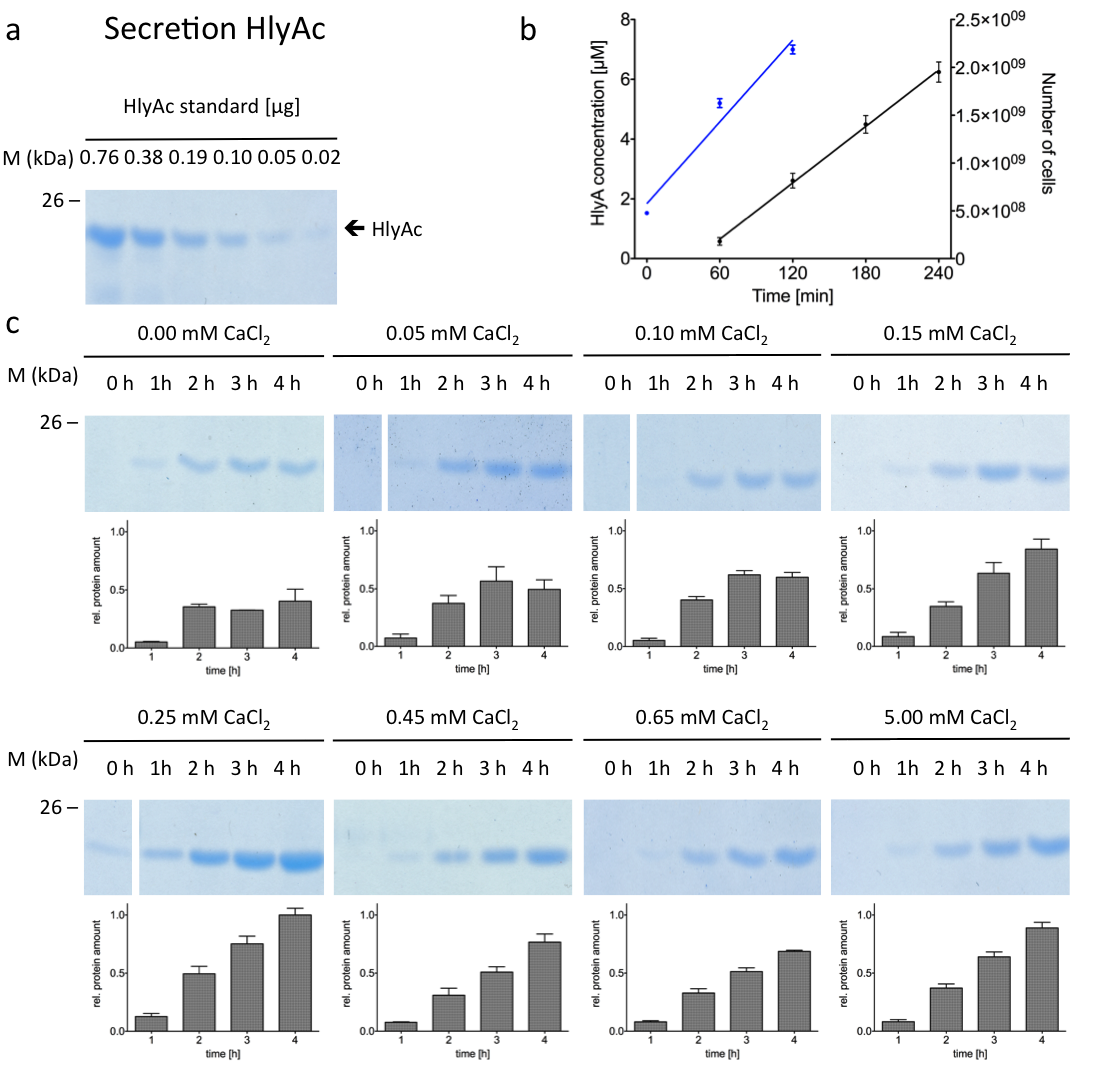


Supplementary Fig. 4


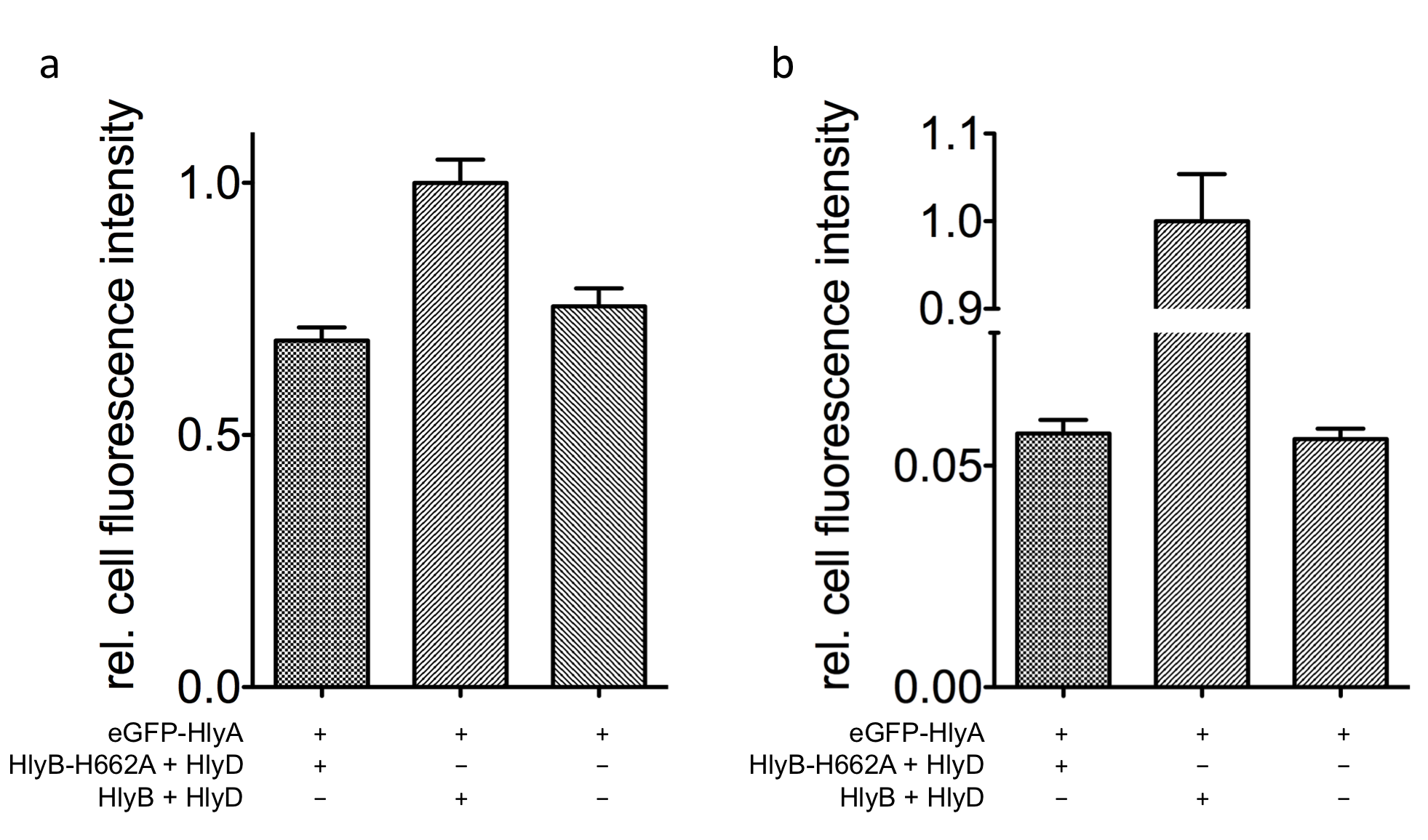


Supplementary Fig. 5


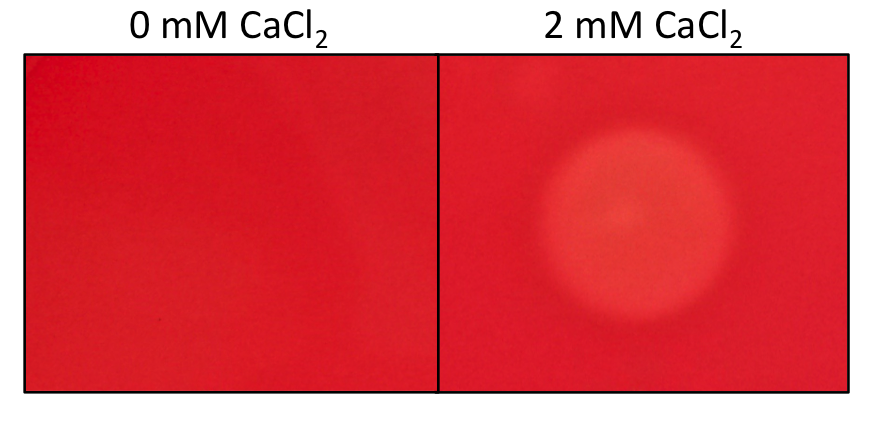


Supplementary Fig. 6


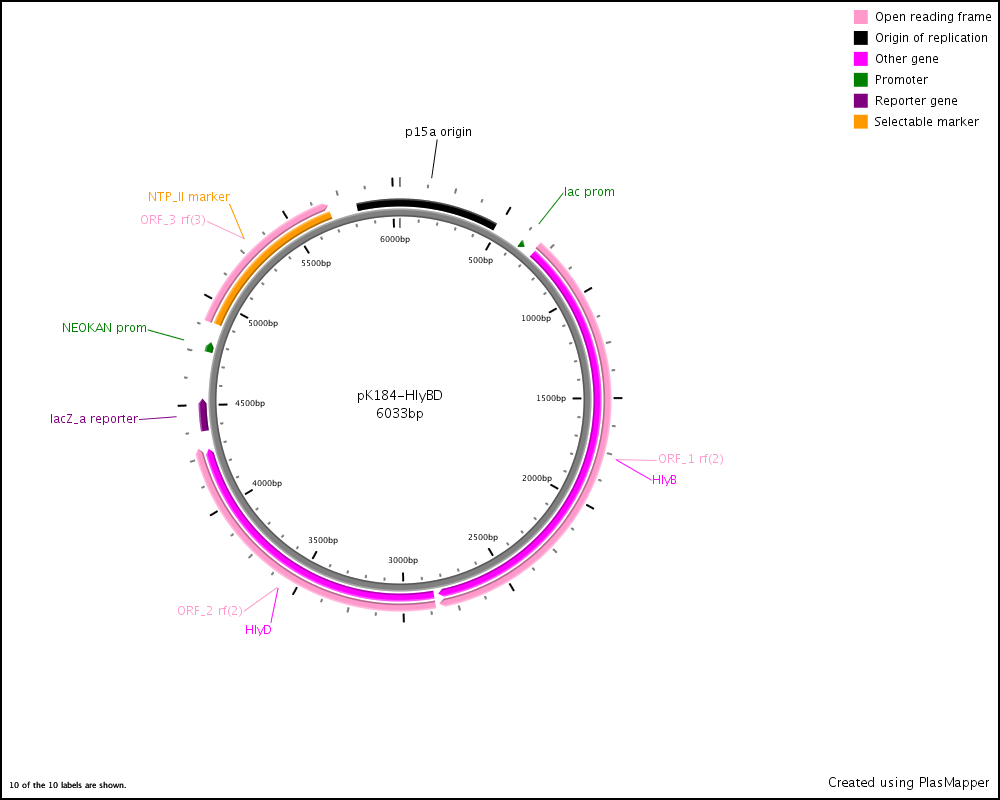


Supplementary Fig. 7


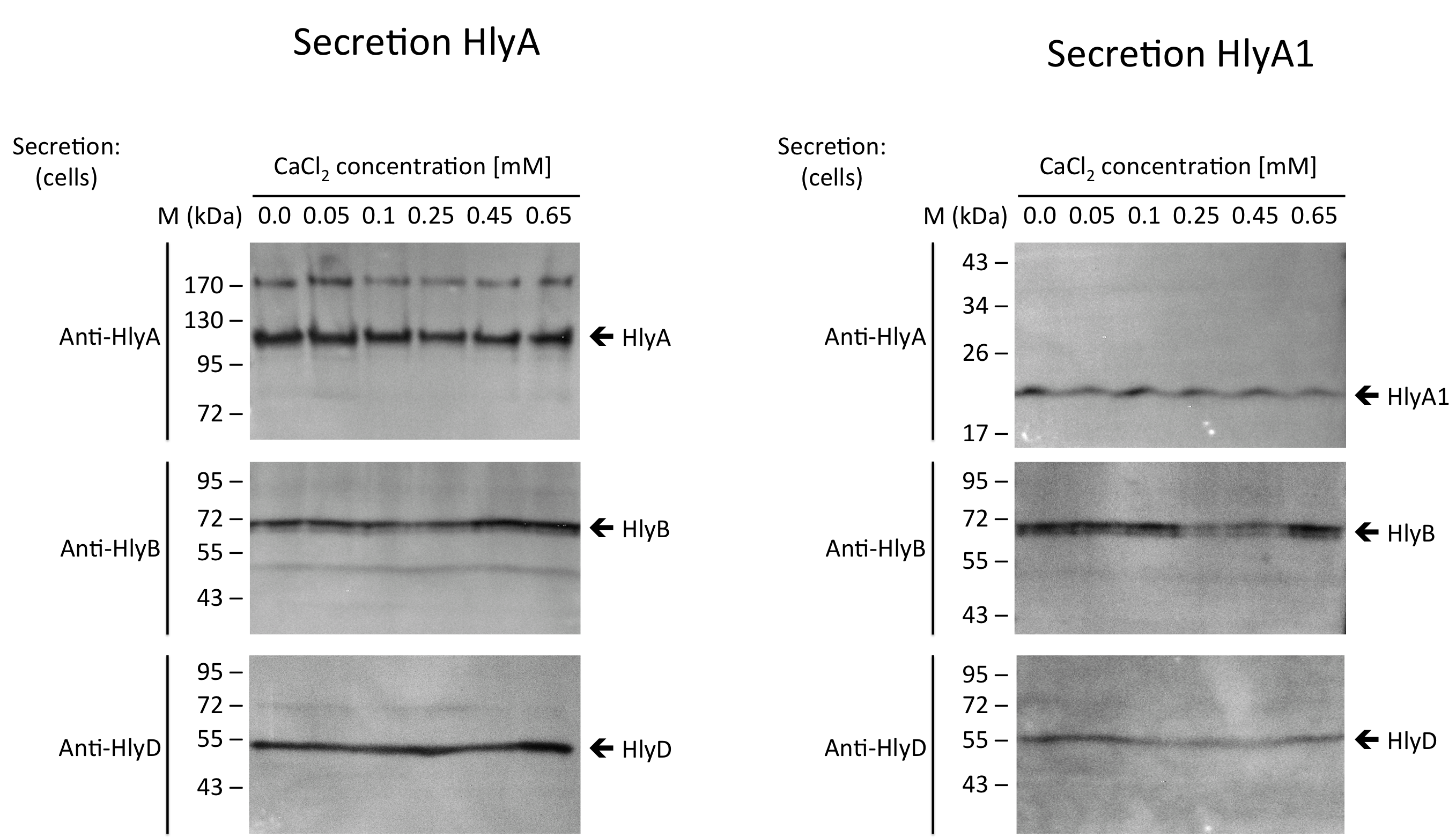


Supplementary Fig. 8
